# Safety of short versus extended antibiotic therapy for neutropenic fever after hematopoietic cell transplantation

**DOI:** 10.1017/ash.2026.10327

**Published:** 2026-03-23

**Authors:** Lucy Cai, Leah Puglisi, Kyle C. Molina, Miguel Goicoechea

**Affiliations:** 1 Scripps Green Hospital, Torrey Pines, CA, USA; 2 Scripps Health, La Jolla, CA, USA

## Abstract

**Objective:**

To determine safety and effectiveness of short (≤7 d) versus extended (>7 d) antibiotic courses for neutropenic fever in HCT recipients.

**Design:**

Retrospective cohort study.

**Setting:**

Private tertiary referral center.

**Participants:**

Consecutive sample; all patients >18 years old admitted between January 2019 and May 2024 with neutropenic fever during their index hospitalization for HCT.

**Methods:**

Data were collected via chart review. Primary outcomes were clinical failure (30-day mortality or ICU admission) and adverse events (*Clostridioides difficile* infection or acute kidney injury [AKI]). Secondary outcomes included recurrent fever and length of stay (LOS). Multivariable logistic regression adjusted for age, sex, transplant type, infection type, and malignancy.

**Results:**

Among 103 patients (55 short, 48 extended), mean antibiotic duration was 3.6 days (short) and 11.9 days (extended). There were more leukemia and allogeneic HCT recipients in the extended group. In multivariable analyses, antibiotic duration was not predictive of clinical failure (odds ratio [OR] 3.40; 95% confidence interval [CI], 0.50–27.55; *P* = .222) or composite adverse events (OR, 3.72; 95% CI, 0.94–16.22; *P* = .067), although the odds of AKI were greater in the extended group (OR, 4.72; 95% CI, 1.08–23.68; *P* = .046). Recurrent fever was uncommon. LOS was greater in the extended group (43.4 d vs 21.4 d; *P* = .032).

**Conclusions:**

We found that shorter antibiotic courses were not associated with worse clinical outcomes or adverse events in HCT patients with neutropenic fever in the early posttransplant period. These findings support emerging evidence favoring shorter therapy.

## Introduction

Neutropenic fever is a frequent and potentially serious complication among patients with hematologic malignancies, affecting 60%–90% of individuals undergoing chemotherapy or hematopoietic cell transplantation (HCT).^1^ The optimal duration of empiric antibiotic therapy for neutropenic fever is unknown. Guidelines on antibiotic de-escalation in the setting of neutropenic fever vary. The 2010 Infectious Diseases Society of America (IDSA) guidelines recommends continued antibiotic therapy until neutrophil recovery, while the 2011 European Conference on Infections in Leukemia (ECIL-4) guidelines supports early discontinuation of antibiotics in patients who have been hemodynamically stable and afebrile for 48 hours, regardless of neutrophil count.^2,3^ In practice, many clinicians are reluctant to stop antibiotics during ongoing neutropenia, despite emerging evidence that shorter courses may be safe.^4,5^ Prolonged antibiotic use increases the risk of *Clostridioides difficile* infection, antimicrobial resistance, and graft-versus-host disease (GvHD) in HCT recipients.^6–9^

While some studies have evaluated outcomes in patients with neutropenic fever who undergo early versus late antibiotic de-escalation, few have examined outcomes in patients receiving antibiotics for set durations. The ANTIBIOSTOP trial found that patients prescribed a short set course of antibiotics of 5 days had significantly less antibiotic exposure compared to patients placed on ECIL-4 protocol without differences in unfavorable outcomes such as mortality, intensive care unit (ICU) admission, and relapsed infection.^10^ A recent randomized controlled trial also found that short-course carbapenem therapy was non-inferior to extended therapy with respect to treatment failure.^11^ Even fewer studies have focused exclusively on HCT recipients with neutropenic fever during the immediate posttransplant period. We identified only one other study studying only HCT recipients with neutropenic fever,^12^ which found that stopping antibiotics following five days of empiric therapy and defervescence was safe. Given the high risk of morbidity and mortality in this population and the limited data on optimal antibiotic durations for these patients, our retrospective study aimed to evaluate whether short-course antibiotic therapy is as safe and effective as extended-course therapy for HCT recipients that develop neutropenic fever in the immediate posttransplant period.

## Methods

### Design

We performed a single-center retrospective cohort study. Our primary objective was to determine if there was a significant difference in clinical failure, defined as ICU admission or absolute 30-day mortality, between patients who received short compared to extended courses of antibiotics for neutropenic fever during their index hospitalization after HCT. Short courses of antibiotics were defined as 7 days or less of antibiotics and extended courses of antibiotics as greater than 8 days of antibiotics.

### Inclusion and exclusion

Patients who received hematopoietic cell transplant between January 1^st^, 2019, and May 1^st^, 2024, at Scripps Green Hospital were identified from the hospital transplant registry. Demographics, clinical data, and transplant details were extracted from electronic health records. Patients over the age of 18 years old who met criteria for neutropenic fever following the date of their hematopoietic cell transplant were included. Consistent with IDSA criteria,^3^ neutropenic fever was defined as an absolute neutrophil count <500 cells/µL with either a single oral temperature >38.3 °C or a temperature >38.0 °C sustained for at least 1 hour.

Only initial, rather than recurrent, febrile episodes were assessed. Febrile episodes, or cases, were classified as:

- **Confirmed infection:** Positive cultures or molecular assays and imaging or exam findings indicative of infection, as documented by the treating physician.
- **Suspected infection:** Imaging, laboratory, or exam findings suggestive of infection as documented by the treating physician without positive cultures or molecular assays (e.g. findings of “colitis” on imaging studies was considered suspected infection).
- **Fever without evidence of infection:** Absence of positive cultures, molecular assays, or findings suggestive of infection as documented by the treating physician. “Oral mucositis” alone was not considered suspected infection unless superimposed infection was documented.

Only index hospitalizations for the initial hematopoietic cell transplant were included to avoid over-representation of a small subset of patients with multiple admissions and prolonged immunosuppression. Episodes of neutropenic fever during prior or subsequent hospitalizations were not included, except for fevers that met criteria for recurrent fever. The study was approved by the Scripps Institutional Review Board.

### Endpoints

The first primary endpoint was clinical failure, a composite endpoint of ICU admission, and all-cause 30-day mortality. The second primary endpoint was adverse events, a composite endpoint of *C. difficile* infection and acute kidney injury (AKI) that occurred after the onset of neutropenic fever during the hospitalization. Secondary endpoints included recurrent fever, defined as relapse of fever after 48 hours afebrile and within seven days of stopping antibiotics, and length of hospital stay. Length of hospital stay (LOS) was categorized as shorter (≤50th percentile) or longer (>50th percentile). While clinically significant relapses resulting in mortality or ICU admission were reflected in our primary endpoint of clinical failure, recurrent fever was included as a secondary endpoint given it is a frequent reason clinicians are hesitant to de-escalate antibiotics or will re-escalate antibiotics shortly after discontinuation.

### Statistical analysis

To compare demographic, transplant, and clinical characteristics between the short and extended antibiotic groups, categorical variables were summarized as frequencies and percentages. Comparisons between groups were made using *χ* ^2^ tests or Fisher’s exact tests. Continuous variables were summarized as means and standard deviations if normally distributed and compared using *t*-tests; otherwise, they were summarized as medians and interquartile ranges and compared using Mann–Whitney *U* tests. Univariable logistic regression models were used to estimate odds ratios and confidence intervals for primary and secondary outcomes between the short and extended antibiotic groups. Multivariable logistic regression models were used to analyze primary and secondary outcomes, adjusting for covariables including age, Hematopoietic Cell Transplantation-specific Comorbidity Index (HCT-CI) score, sex, ethnicity, race, type of transplant, type of hematologic malignancy, type of febrile case, and year of admission. Stratified analyses were conducted using univariable logistic regression and χ^2^ tests. Two-tailed *p*-values less than .05 were considered statistically significant. All analyses were performed using R Studio (version 4.3.3, RStudio, 2025).

## Results

### Baseline characteristics

During the study period, 206 patients received a total of 211 hematopoietic cell transplants. Five patients had received more than one transplant. 103 patients met inclusion criteria. Two patients had undergone more than one transplant, but both patients had neutropenic fever only during one of their index hospitalizations. Baseline characteristics are summarized in Table 1.

**Table 1.** Baseline characteristics for patients who received short and extended antibiotic therapy. Categorical data is reported as counts with percentages in parentheses, and numeric data is reported as means with standard deviations in parentheses. A *p*-value < .05 was considered statistically significant

Item	Antibiotic duration		*p*-value
	Short (*n* = 55)	Extended (*n* = 48)	
Age at admission (years)	56 (12.4)	53 (13.7)	.169
Sex			
* Female*	18 (32.7%)	14 (29.2%)	.697
* Male*	37 (67.3%)	34 (70.8%)	
Type of transplant			
* Allogeneic*	13 (23.6%)	27 (56.3%)	.001
* Autologous*	42 (76.4%)	21 (43.8%)	
Race			.845
* Asian*	5 (9.1%)	5 (10.4%)	
* Black*	1 (1.8%)	2 (4.2%)	
* Native Hawaiian or Other Pacific Islander*	1 (1.8%)	0 (0%)	
* White*	47 (85.5%)	39 (81.3%)	
* Not Reported*	1 (1.8%)	2 (4.2%)	
Ethnicity			.479
* Hispanic or Latino or Spanish Origin*	12 (21.8%)	14 (29.2%)	
* Not Hispanic, Latino/a, or Spanish Origin*	42 (76.4%)	32 (66.7%)	
* Not Reported*	1 (1.8%)	2 (4.2%)	
HCT-CI^1^ score	1.95 (1.81)	2.40 (1.61)	.188
Underlying malignancy			.002
* Leukemias*	7 (12.7%)	21 (43.8%)	<.001
* Lymphomas*	20 (36.4%)	16 (33.3%)	.748
* Myeloproliferative/myelodysplastic disorders*	6 (10.9%)	3 (6.3%)	.498
* Plasma cell disorders*	20 (36.4%)	6 (12.5%)	.005
* Solid tumors*	2 (3.6%)	2 (4.2%)	>.999
Body mass index, kg/m^2^	29.4 (5.65)	27.9 (5.67)	.205
Year of admission			.150
* 2019*	15 (27.3%)	8 (16.7%)	
* 2020*	10 (18.2%)	9 (18.8%)	
* 2021*	13 (23.6%)	7 (14.6%)	
* 2022*	12 (21.8%)	13 (27.1%)	
* 2023*	4 (7.3%)	11 (22.9%)	
* 2024*	1 (1.8%)	0 (0%)	

1 *HCT-CI;* Hematopoietic Cell Transplantation-specific Comorbidity Index.

Characteristics were generally similar between the two groups, except for transplant type and type of malignancy. The short group had a higher percentage of autologous transplants compared to the extended antibiotic group (76.4% vs 43.8%; *P* = .001). There was a higher percentage of leukemia (43.8% vs 12.7%; *P* < .001) and lower percentage of plasma cell dyscrasia patients (12.5% vs 36.4%; *P* = .005) in the extended group compared to the short group. Mean antibiotic duration was 3.6 versus 11.9 days in short and extended groups, respectively. The absolute neutrophil count at the onset of neutropenic fever, time to engraftment, and HCT-Cl score were similar between the two groups.

Confirmed (43.8% vs 5.5%) and suspected infection (35.4% vs 10.9%) were more common in the extended compared to the short group (*P* < .001). The extended antibiotic group had a small but significantly longer duration of fever compared to the short group (2.73 vs 1.45 d; *P* = .012), more frequent septic shock (18.8% vs 1.8%; *P* = .005), and higher rates of bacteremia (41.7% vs 3.6%; *P* < .001) (Table 2). There was significant overlap between confirmed infections and bacteremia, with over 80% of confirmed infections being bacteremia.

**Table 2.** Additional markers of illness severity. Categorical data is reported as counts with percentages in parentheses, and numeric data is reported as means with standard deviations in parentheses. A *p*-value < .05 was considered statistically significant

Item	Antibiotic duration		*p*-value
	Short (*n* = 55)	Extended (*n* = 48)	
Bacteremia	2 (3.6%)	20 (41.7%)	<.001
Septic shock	1 (1.8%)	9 (18.8%)	.005
Duration of fever	1.45 (0.74)	2.73 (3.32)	.012
ANC^1^ at fever onset	42.55 (89.6)	19.79 (47.9)	.106
Duration of antibiotics (days)	3.56 (1.77)	11.88 (5.78)	–
Time to engraftment (days)	15.23 (25.36)	14.16 (4.47)	.781
Type of febrile case			<.001
* Confirmed infection*	3 (5.5%)	21 (43.8%)	<.001
* Suspected infection*	6 (10.9%)	17 (35.4%)	.003
* Fever without evidence of infection*	46 (83.6%)	10 (20.8%)	<.001

1 *ANC*; absolute neutrophil count (K/µL).

A total of 14 different species were identified on blood cultures. Distribution of bacterial species was similar except for *Escherichia coli*, which was more common in the extended group (21% vs 2%; *P* = .003). Other genera of bacteria (and number of isolates) included *Enterococci* (2), *Streptococci* (2), *Klebsiella* (1), *Citrobacter* (1), *Staphylococci* (2), *Pseudomonas* (2), *Leptotrichia* (1), *Gemella* (1), and *Rothia* (1). Six patients had been treated for presumed or confirmed fungal infections. Four patients had tested positive for respiratory viruses via molecular assays (respiratory syncytial virus, parainfluenza 1, and COVID-19). Vancomycin, ceftriaxone, and meropenem were more frequently used in the extended group (all *P* < .010). There was no significant difference in the use of piperacillin-tazobactam, daptomycin, metronidazole, trimethoprim-sulfamethoxazole, or cefepime between the two groups.

### Primary endpoints

A summary of results is presented in Table 3. The unadjusted rate of clinical failure was more frequent in the extended compared to the short group (35.4% vs 9.1%; *P* = .001). Both confirmed (OR, 6.56; 95% CI, 1.96–24.45; *P* = .003) and suspected infections (OR, 5.1; 95% CI, 1.49–19.05; *P* = .011) were significant predictors of clinical failure compared to fever with no evidence of infection in the univariable logistic regression. Two deaths occurred in the short group, with five deaths in the extended group. In the multivariable analysis, antibiotic duration did not predict clinical failure (OR, 3.40; 95% CI, 0.50–27.55; *P* = .222) or ICU admission (OR, 5.32; 95% CI, 0.64–58.06; *P* = .136).

**Table 3.** Summary of primary and secondary outcomes. Categorical data is reported as counts with percentages in parentheses, and numeric data is reported as means with standard deviations in parentheses. Multivariable logistic regression adjusted for variables described in the methods section. A *p*-value < .05 was considered statistically significant

	Antibiotic duration		*p*-value		
Item	Short	Extended	Univariable analysis	Univariable LR^1^	Multivariable LR
**Primary outcomes**					
*Clinical failure*, *n* (%)					
All-cause mortality	2 (3.6%)	5 (10.4%)	0.247	–	–
ICU^2^ admission	5 (9.1%)	16 (33.3%)	0.002	.004	.136
Clinical failure (composite)	5 (9.1%)	17 (35.4%)	0.001	.002	.222
*Adverse events*, *n* (%)					
Acute kidney injury	7 (12.7%)	21 (43.8%)	<0.001	.004	.046
*Clostridioides difficile* infection	1 (1.8%)	5 (10.4%)	0.095	–	–
Adverse events (composite)	8 (14.5%)	24 (50.0%)	<0.001	<.001	.067
**Secondary outcomes**					
Recurrent fever, *n* (%)	2 (3.6%)	3 (6.3%)	0.662	–	–
Length of hospitalization, days, mean (SD)^3^	21.35 (9.29)	43.44 (68.97)	0.032	<.001	.045

1 *LR*; logistic regression.

2 *ICU*; intensive care unit.

3 *SD*; standard deviation.

To further control for the effect of the type of fever on the relationship between clinical failure and antibiotic duration, we performed a stratified analysis using *χ* ^2^ tests or Fisher’s exact tests. Clinical failure rates between the extended and short groups did not differ significantly within each strata, which was confirmed with logistic regression. Antibiotic duration did not predict clinical failure for HCT recipients with fever without evidence of infection (OR, 3.58; 95% CI, 0.42–25.24; *P* = .198), suspected infection (OR, 4.40; 95% CI, 0.55–95.04; *P* = .213), or confirmed infection (OR, 1; 95% CI, 0.08–23.72; *P* > .999).

Unadjusted composite adverse events were more common in the extended group compared to the short group (50% vs 14.5%; *P* < .001). *C. difficile* infection rates were greater in the extended group (10.4%) than the short group (1.8%), although the difference was not significant (*P* = .095). When adjusted for covariables, antibiotic duration was a near but not a significant predictor of composite adverse events (OR, 3.72; 95% CI, 0.94–16.22; *P* = .067). However, the extended group had higher rates of AKI (43.8% vs 12.7%; *P* < .001). In the multivariable analysis, those who received extended therapy were still more likely to experience AKI compared to those who received a short course (OR, 4.72; 95% CI, 1.08–23.68; *P* = .046).

### Secondary endpoints

There was no significant difference in recurrent fever between the short and extended groups (6.3% vs 3.6%, *P* = .662). Recurrent fever was relatively uncommon, with only five episodes of recurrent fever (two in the short group and three in the extended group). The median length of hospital stay (LOS) was 21 days (IQR 18–29), so a shorter antibiotic stay (less than or equal to 50^th^ percentile) was defined as less than 22 days and a longer hospital stay (>50^th^ percentile) was defined as 22 days or greater. LOS was longer in the extended group compared to the short group (43.4 d vs 21.4 d; *P* = .032). Antibiotic duration, age, type of malignancy, type of febrile case, and type of transplant predicted a longer hospital stay in the univariable logistic regression. In the multivariable analysis, only antibiotic duration remained predictive of a longer length of stay (LOS) (OR, 83.4; 95% CI, 2.74–38,294; *P* = .045).

## Discussion

While recent studies have shown shorter antibiotic courses are safe, overall data is still scarce. To our knowledge, our study is one of the few to evaluate short course antibiotic therapy for neutropenic fever exclusively in hematopoietic cell recipients. We found that shorter antibiotic therapy does not lead to greater clinical failure in HCT recipients with undifferentiated neutropenic fever. Specifically, our findings in patients with neutropenic fever and no identifiable source of infection align with recent meta-analyses and systematic reviews, which have found no link between early discontinuation of antibiotics and an increased risk of serious outcomes.^13–15^

Previous studies have generally excluded patients with documented or suspected infections. In contrast, we included all patients with neutropenic fever, regardless of whether an infection was confirmed or suspected. As expected, patients in our study with confirmed or suspected infections were more likely to experience clinical failure, which is consistent with prior research showing that confirmed infections are known risk factors for mortality.^16^ Bloodstream infection is likely an independent risk factor for poor outcomes, regardless of antibiotic duration. Our stratified analyses suggest that shorter courses of antibiotics are not associated with worse clinical outcomes compared to extended courses, even for patients with documented or suspected infections. However, most of our patients with confirmed infections had gram-negative bacteremia. For non-neutropenic patients, a seven-day course of antibiotics is often sufficient for uncomplicated gram-negative bacteremia,^17^ although further evaluation is needed to determine the optimal duration in HCT recipients with neutropenic fever. We had only a small number of patients with gram-positive or fungal infections and were therefore unable to evaluate these subgroups. Further research is needed to determine the optimal antibiotic duration for infections caused by different organisms in this population.

Shorter course antibiotic therapy tended to have fewer antibiotic-associated adverse events such as AKI and *C. difficile* infections. AKI and longer hospital stays were more common among the extended group. However, patients receiving extended therapy also had a greater frequency of bacteremia and septic shock, which likely increased their risk of acute tubular necrosis and prolonged their hospital stays. As a result, extended antibiotic therapy may not have been the only risk factor for AKI. The short group also had a reduced rate of *C. difficile* infections compared to the extended group (1.8% vs 10.4%), although this difference was not statistically significant.

Our study has several limitations. Our retrospective design made it difficult to eliminate all confounders. Although we controlled for the effects of confirmed and suspected infection on clinical failure, heterogeneity likely exists in clinicians’ diagnostic and treatment approaches. Patients could not be randomized to set durations of antibiotics, and no universal practice guidelines for antibiotic de-escalation or duration were applied. Compared to patients who received a short antibiotic course, those who received an extended antibiotic course were also more likely to have bacteremia, septic shock, and to have received multiple antibiotic classes, suggesting an overall greater severity of illness. The extended group also included more patients with leukemia and allogeneic HCT; despite adjustment for transplant type and malignancy, residual differences may limit generalizability to higher-risk HCT populations. In addition, we only included index hospitalizations in our analyses, thus decreasing generalizability to subsequent hospitalizations for neutropenic fever. Our study is limited by a modest sample size of 103 patients; however, it remains one of the larger cohorts focused exclusively on neutropenic fever in the immediate posttransplant period. Nevertheless, larger multicenter studies are warranted to confirm these results and further define optimal antibiotic durations in this vulnerable population. While we did not find a significant difference in mortality, *C. difficile* infections, or recurrent fever between the two groups, the lack of significance may be due to the overall low frequency of these events.

In conclusion, our results are consistent with prior studies that have shown that short-term antibiotic therapy does not lead to higher rates of clinical failure compared to long-term antibiotic therapy for neutropenic fever. We suggest that even in the immediate posttransplant period, a shorter antibiotic course is not associated with significantly worse outcomes, particularly in patients without documented infections and autologous HCT recipients. Shorter antibiotic courses may also reduce the risk of AKI and *C. difficile* infection. However, larger randomized studies are needed to confirm that shorter antibiotic courses are safe in this vulnerable population and elucidate the optimal antibiotic durations for patients with suspected or documented infections.

## Supporting information

10.1017/ash.2026.10327.sm001 Cai et al. supplementary material Cai et al. supplementary material

## Supplementary material

The supplementary material for this article can be found at https://doi.org/10.1017/ash.2026.10327

## Research transparency and reproducibility

The data set and codes are not publicly available but are available from the corresponding author on reasonable request.
