# Supplementary material for: Safety of short versus extended antibiotic therapy for neutropenic fever after hematopoietic cell transplantation
Source: Antimicrob Steward Healthc Epidemiol. 2026 Mar 23;6(1):e58. doi: 10.1017/ash.2026.10327 (PMC13104521; doi:10.1017/ash.2026.10327)
Supplement: Cai et al. supplementary material [file S2732494X26103271sup001.docx]

**Supplemental Material**

|  | **Univariable analysis** | | | | **Multivariable analysis** | | | |
| --- | --- | --- | --- | --- | --- | --- | --- | --- |
| **Item** | **OR** | **95% CI**^1^ | | ***p*-value** | **OR**^2^ | **95% CI** | | ***p*-value** |
| HCT-CI^3^ score | 1.05 | 0.80 | 1.37 | 0.717 |  | | | |
| Age | 0.98 | 0.95 | 1.02 | 0.27 |  | | | |
| Antibiotic duration | | | | | | | | |
| *0-7 days* | Reference | - | - | - |  | | | |
| *8 or more days* | 5.48 | 1.95 | 18.06 | 0.002 | 3.4 | 0.5 | 27.55 | 0.222 |
| Sex | | | | | | | | |
| *Female* | Reference | - | - | - |  | | | |
| *Male* | 0.96 | 0.36 | 2.77 | 0.932 |  | | | |
| Race | | | | | | | | |
| *Not White* | Reference | - | - | - |  | | | |
| *White* | 0.42 | 0.14 | 1.37 | 0.133 |  | | | |
| Ethnicity | | | | | | | | |
| *Hispanic* | Reference | - | - | - |  | | | |
| *Non-Hispanic* | 1.16 | 0.39 | 3.89 | 0.797 |  | | | |
| Underlying malignancy | | | | | | | | |
| *Leukemia* | Reference | - | - | - |  | | | |
| *Lymphoma* | 0.71 | 0.23 | 2.25 | 0.561 |  | | | |
| *Myeloproliferative or myelodysplastic syndromes* | 3.13 | 0.67 | 15.76 | 0.149 |  | | | |
| *Plasma cell disorders* | 0 | NA | INF | 0.989 |  | | | |
| *Solid tumor* | 0.83 | 0.04 | 7.70 | 0.882 |  | | | |
| Type of transplant | | | | | | | | |
| *Allogeneic* | Reference | - | - | - |  | | | |
| *Autologous* | 0.21 | 0.07 | 0.56 | 0.002 |  | | | |
| Year of transplant | | | | | | | | |
| *2019* | Reference | - | - | - |  | | | |
| *2020* | 0.76 | 0.17 | 3.16 | 0.703 |  | | | |
| *2021* | 0.50 | 0.09 | 2.23 | 0.378 |  | | | |
| *2022* | 0.39 | 0.07 | 1.69 | 0.221 |  | | | |
| *2023/2024* | 1.70 | 0.43 | 6.91 | 0.449 |  | | | |
| Type of febrile case at antibiotic initiation | | | | | | | | |
| *Fever without evidence of infection* | Reference | - | - | - |  | | | |
| *Suspected infection* | 6.56 | 1.96 | 24.45 | 0.003 |  | | | |
| *Confirmed infection* | 5.1 | 1.49 | 19.05 | 0.011 |  | | | |

**Table S1.** Clinical failure for short compared to extended antibiotic therapy, univariable and multivariable analyses using logistic regression. Multivariable analyses adjusted for variables described in the Methods section. A *p*-value <0.05 was considered statistically significant.

^1^*CI*; confidence interval

^2^*OR*; odds ratio

^3^*HCT-CI;* Hematopoietic Cell Transplantation-specific Comorbidity Index

|  | **Antibiotic Duration** | | | | ***p*-value** |
| --- | --- | --- | --- | --- | --- |
|  | **Short** | | **Extended** | |  |
| **Type of febrile case** | Frequency (*n*) | Percentage (%) | Frequency (*n*) | Percentage (%) |  |
| Confirmed Infection | 1 | 33.3 | 7 | 33.3 | >0.999 |
| Suspected Infection | 1 | 16.7 | 8 | 47.1 | 0.340 |
| Without evidence of infection | 3 | 6.5 | 2 | 20 | 0.214 |

**Table S2a**. Association between antibiotic duration and clinical failure, stratified by type of febrile case using chi-squared or Fisher’s exact test. A *p*-value <0.05 was considered statistically significant.

|  |  | | | |
| --- | --- | --- | --- | --- |
| **Type of febrile case** | **OR** | **95% CI** | | ***p*-value** |
| Confirmed Infection | 1 | 0.08 | 23.72 | >0.999 |
| Suspected Infection | 4.4 | 0.55 | 95.04 | 0.213 |
| Without evidence of infection | 3.58 | 0.42 | 25.24 | 0.198 |

**Table S2b**. Association between antibiotic duration and clinical failure, stratified by type of febrile case. Univariable logistic regression was used to compare short versus extended antibiotic duration. Odds ratios (OR) reflect the likelihood of clinical failure with short antibiotic duration. A *p*-value <0.05 was considered statistically significant.

|  | **Univariable analysis** | | | | **Multivariable analysis** | | | |
| --- | --- | --- | --- | --- | --- | --- | --- | --- |
| **Item** | **OR** | **95% CI**^1^ | | ***p*-value** | **OR**^2^ | **95% CI** | | ***p*-value** |
| HCT-CI^3^ score | 1.02 | 0.76 | 1.33 | 0.916 |  | | | |
| Age | 0.99 | 0.95 | 1.03 | 0.51 |  | | | |
| Antibiotic duration | | | | | | | | |
| *0-7 days* | Reference | - | - | - |  | | | |
| *8 or more days* | 5 | 1.77 | 16.52 | 0.004 | 5.32 | 0.64 | 58.06 | 0.136 |
| Sex | | | | | | | | |
| *Female* | Reference | - | - | - |  | | | |
| *Male* | 0.88 | 0.32 | 2.55 | 0.802 |  | | | |
| Race | | | | | | | | |
| *Not White* | Reference | - | - | - |  | | | |
| *White* | 0.39 | 0.13 | 1.27 | 0.103 |  | | | |
| Ethnicity | | | | | | | | |
| *Hispanic* | Reference | - | - | - |  | | | |
| *Non-Hispanic* | 1.07 | 0.36 | 3.6 | 0.909 |  | | | |
| Underlying malignancy | | | | | | | | |
| *Leukemia* | Reference | - | - | - |  | | | |
| *Lymphoma* | 0.86 | 0.27 | 2.8 | 0.795 |  |  |  |  |
| *Myeloproliferative or myelodysplastic syndromes* | 3.75 | 0.79 | 19.34 | 0.099 |  |  |  |  |
| *Plasma cell disorders* | 0 | NA | INF | 0.989 |  | | | |
| *Solid Tumor* | 1 | 0.05 | 9.39 | >0.999 |  | | | |
| Type of transplant | | | | | | | | |
| *Allogeneic* | Reference | - | - | - |  | | | |
| *Autologous* | 0.23 | 0.08 | 0.63 | 0.005 |  |  |  |  |
| Year of transplant | | | | | | | | |
| *2019* | Reference | - | - | - |  | | | |
| *2020* | 0.76 | 0.17 | 3.16 | 0.703 |  | | | |
| *2021* | 0.5 | 0.09 | 2.23 | 0.378 |  | | | |
| *2022* | 0.25 | 0.03 | 1.22 | 0.11 |  | | | |
| *2023/2024* | 1.7 | 0.43 | 6.91 | 0.449 |  | | | |
| Type of febrile case at antibiotic initiation | | | | | | | | |
| *Fever without evidence of infection* | Reference | - | - | - |  | | | |
| *Suspected infection* | 6.56 | 1.96 | 24.45 | 0.003 |  | | | |
| *Confirmed infection* | 4.2 | 1.19 | 15.91 | 0.027 |  | | | |

**Table S3.** Intensive care unit admission for short compared to extended antibiotic therapy, univariable and multivariable analyses using logistic regression. Multivariable analyses adjusted for variables described in the Methods section. A *p*-value <0.05 was considered statistically significant.

^1^*CI*; confidence interval

^2^*OR*; odds ratio

^3^*HCT-CI;* Hematopoietic Cell Transplantation-specific Comorbidity Index

|  | **Univariable analysis** | | | | **Multivariable analysis** | | | |
| --- | --- | --- | --- | --- | --- | --- | --- | --- |
| **Item** | **OR** | **95% CI**^1^ | | ***p*-value** | **OR**^2^ | **95% CI** | | ***p*-value** |
| HCT-CI^3^ score | 1.096 | 0.859 | 1.397 | 0.456 |  | | | |
| Age | 1.01 | 0.978 | 1.045 | 0.539 |  | | | |
| Antibiotic duration | | | | | | | | |
| *0-7 days* | Reference | - | - | - |  | | | |
| *8 or more days* | 5.875 | 2.378 | 15.822 | <0.001 | 3.72 | 0.94 | 16.22 | 0.067 |
| Sex | | | | | | | | |
| *Female* | Reference | - | - | - |  | | | |
| *Male* | 0.988 | 0.406 | 2.497 | 0.979 |  | | | |
| Race | | | | | | | | |
| *Not White* | Reference | - | - | - |  | | | |
| *White* | 1.57 | 0.5 | 5.96 | 0.465 |  | | | |
| Ethnicity | | | | | | | | |
| *Hispanic* | Reference | - | - | - |  | | | |
| *Non-Hispanic* | 0.54 | 0.21 | 1.38 | 0.193 |  | | | |
| Underlying malignancy |  |  |  |  |  | | | |
| *Leukemia* | Reference | - | - | - |  | | | |
| *Lymphoma* | 0.29 | 0.1 | 0.82 | 0.022 |  | | | |
| *Myeloproliferative or myelodysplastic syndromes* | 0.11 | 0.01 | 0.7 | 0.048 |  | | | |
| *Plasma cell disorders* | 0.21 | 0.06 | 0.67 | 0.012 |  | | | |
| *Solid Tumor* | 0.87 | 0.09 | 8.07 | 0.894 |  | | | |
| Type of transplant | | | | | | | | |
| *Allogeneic* | Reference | - | - | - |  | | | |
| *Autologous* | 0.42 | 0.18 | 0.99 | 0.048 |  | | | |
| Year of transplant | | | | | | | | |
| *2019* | Reference | - | - | - |  | | | |
| *2020* | 1.65 | 0.44 | 6.38 | 0.455 |  | | | |
| *2021* | 0.94 | 0.23 | 3.77 | 0.935 |  | | | |
| *2022* | 1.59 | 0.47 | 5.73 | 0.461 |  | | | |
| *2023/2024* | 1.29 | 0.3 | 5.34 | 0.725 |  | | | |
| Type of febrile case at antibiotic initiation | | |  |  |  | | | |
| *Fever without evidence of infection* | Reference | - | - | - |  | | | |
| *Suspected infection* | 4.79 | 1.64 | 14.62 | 0.005 |  | | | |
| *Confirmed infection* | 5.22 | 1.82 | 17.78 | 0.003 |  | | | |

**Table S4.** Composite adverse events for short compared to extended antibiotic therapy, univariable and multivariable analyses using logistic regression. Multivariable analyses adjusted for variables described in the Methods section. A *p*-value <0.05 was considered statistically significant.

^1^*CI*; confidence interval

^2^*OR*; odds ratio

^3^*HCT-CI;* Hematopoietic Cell Transplantation-specific Comorbidity Index

|  | **Univariable analysis** | | | | **Multivariable analysis** | | | |
| --- | --- | --- | --- | --- | --- | --- | --- | --- |
| **Item** | **OR** | **95% CI**^1^ | | ***p*-value** | **OR**^2^ | **95% CI** | | ***p*-value** |
| HCT-CI^3^ score | 1.08 | 0.84 | 1.39 | 0.549 |  | | | |
| Age | 1.03 | 0.99 | 1.07 | 0.155 | 1.09 | 1.03 | 1.16 | 0.003 |
| Antibiotic duration | | | | | | | | |
| *0-7 days* | Reference | - | - | - |  | | | |
| *8 or more days* | 5.33 | 2.09 | 15.06 | 0.001 | 4.72 | 1.08 | 23.68 | 0.046 |
| Sex | | | | | | | | |
| *Female* | Reference | - | - | - |  | | | |
| *Male* | 1.18 | 0.46 | 3.18 | 0.738 |  | | | |
| Race | | | | | | | | |
| *Not White* | Reference | - | - | - |  | | | |
| *White* | 1.26 | 0.4 | 4.81 | 0.711 |  | | | |
| Ethnicity | | | | | | | | |
| *Hispanic* | Reference | - | - | - |  | | | |
| *Non-Hispanic* | 0.65 | 0.25 | 1.75 | 0.384 |  | | | |
| Underlying malignancy | | | | | | | | |
| *Leukemia* | Reference | - | - | - |  | | | |
| *Lymphoma* | 0.38 | 0.12 | 1.11 | 0.081 |  | | | |
| *Myeloproliferative or myelodysplastic syndromes* | 0.17 | 0.01 | 1.09 | 0.112 |  | | | |
| *Plasma cell disorders* | 0.32 | 0.09 | 1.04 | 0.067 |  | | | |
| *Solid Tumor* | 1.33 | 0.14 | 12.46 | 0.788 |  | | | |
| Type of transplant | | | | | | | | |
| *Allogeneic* | Reference | - | - | - |  | | | |
| *Autologous* | 0.53 | 0.22 | 1.28 | 0.158 |  | | | |
| Year of transplant | | | | | | | | |
| *2019* | Reference | - | - | - |  | | | |
| *2020* | 2.1 | 0.54 | 8.63 | 0.285 |  | | | |
| *2021* | 1.2 | 0.28 | 5.1 | 0.801 |  | | | |
| *2022* | 1.4 | 0.38 | 5.52 | 0.618 |  | | | |
| *2023/2024* | 1.2 | 0.25 | 5.47 | 0.812 |  | | | |
| Type of febrile case at antibiotic initiation | | | | | | | | |
| *Fever without evidence of infection* | Reference | - | - | - |  | | | |
| *Suspected infection* | 4.62 | 1.53 | 14.54 | 0.007 |  | | | |
| *Confirmed infection* | 4.29 | 1.43 | 13.34 | 0.010 |  | | | |

**Table S5.** Acute kidney injury (AKI) for short compared to extended antibiotic therapy, univariable and multivariable analyses using logistic regression. Multivariable analyses adjusted for variables described in the Methods section. A *p*-value <0.05 was considered statistically significant.

^1^*CI*; confidence interval

^2^*OR*; odds ratio

^3^*HCT-CI;* Hematopoietic Cell Transplantation-specific Comorbidity Index

|  | **Univariable analysis** | | | | **Multivariable analysis** | | | |
| --- | --- | --- | --- | --- | --- | --- | --- | --- |
| **Item** | **OR** | **95% CI**^1^ | | ***p*-value** | **OR**^2^ | **95% CI** | | ***p*-value** |
| HCT-CI^3^ score | 1.13 | 0.9 | 1.43 | 0.3 |  | | | |
| Age | 0.96 | 0.93 | 0.99 | 0.023 |  | | | |
| Antibiotic duration | | | | | | | | |
| *0-7 days* | Reference | - | - | - |  | | | |
| *8 or more days* | 8 | 3.4 | 20.04 | <0.001 | 83.40 | 2.74 | 38294 | 0.045 |
| Sex | | | | | | | | |
| *Female* | Reference | - | - | - |  | | | |
| *Male* | 1.17 | 0.51 | 2.71 | 0.719 |  | | | |
| Race | | | | | | | | |
| *Not White* | Reference | - | - | - |  | | | |
| *White* | 1.05 | 0.27 | 4.02 | 0.944 |  | | | |
| Ethnicity | | | | | | | | |
| *Hispanic* | Reference | - | - | - |  | | | |
| *Non-Hispanic* | 0.85 | 0.29 | 2.42 | 0.757 |  | | | |
| Underlying malignancy | | | | | | | | |
| *Leukemia* | Reference | - | - | - |  | | | |
| *Lymphoma* | 0.02 | 0 | 0.09 | <0.001 |  |  |  |  |
| *Myeloproliferative or myelodysplastic syndromes* | INF | 0 | NA | 0.994 |  |  |  |  |
| *Plasma cell disorders* | 0 | 0 | 0.02 | <0.001 |  | | | |
| *Solid Tumor* | 0.04 | 0 | 0.54 | 0.021 |  | | | |
| Type of transplant | | | | | | | | |
| *Allogeneic* | Reference | - | - | - |  | | | |
| *Autologous* | 0.01 | 0 | 0.03 | <0.001 |  |  |  |  |
| Year of transplant | | | | | | | | |
| *2019* | Reference | - | - | - |  | | | |
| *2020* | 0.91 | 0.25 | 3.18 | 0.879 |  | | | |
| *2021* | 1.27 | 0.38 | 4.36 | 0.697 |  | | | |
| *2022* | 2.33 | 0.74 | 7.67 | 0.152 |  | | | |
| *2023/2024* | 3.42 | 0.92 | 14.15 | 0.074 |  | | | |
| Type of febrile case at antibiotic initiation | | | | | | | | |
| *Fever without evidence of infection* | Reference | - | - | - |  | | | |
| *Suspected infection* | 3.28 | 1.22 | 9.29 | 0.021 |  | | | |
| *Confirmed infection* | 8.02 | 2.74 | 27.42 | <0.001 |  | | | |

**Table S6.** Length of hospital stay for short compared to extended antibiotic therapy, univariable and multivariable analyses using logistic regression. Multivariable analyses adjusted for variables described in the Materials & Methods section. A *p*-value <0.05 was considered statistically significant.

^1^*CI*; confidence interval

^2^*OR*; odds ratio

^3^*HCT-CI;* Hematopoietic Cell Transplantation-specific Comorbidity Index
